# Eye movements and eating disorders: protocol for an exploratory experimental study examining the relationship in young-adult women with subclinical symptomatology

**DOI:** 10.1186/s40337-022-00573-2

**Published:** 2022-04-08

**Authors:** Sergio Navas-León, Milagrosa Sánchez-Martín, Ana Tajadura-Jiménez, Lize De Coster, Mercedes Borda-Más, Luis Morales

**Affiliations:** 1 Department of Psychology, Universidad Loyola Andalucía, Sevilla, Spain; 2 DEI Interactive Systems Group, Department of Computer Science and Engineering, Universidad Carlos III de Madrid, Leganés, Spain; 3 UCL Interaction Centre (UCLIC), University College London, University of London, London, UK; 4 Department of Psychology, Universidad de Sevilla, Sevilla, Spain

**Keywords:** Antisaccade, Eating disorders, Inhibitory control, Memory-guided saccade, Prosaccade, Saccades, Subclinical population, Square wave jerks, Visual memory

## Abstract

**Background:**

Recent research indicates that patients with anorexia (AN) show specific eye movement abnormalities such as shorter prosaccade latencies, more saccade inhibition errors, and increased rate of saccadic intrusions compared to participants without AN. However, it remains unknown whether these abnormal eye movement patterns, which may serve as potential biomarkers and endophenotypes for an early diagnosis and preventive clinical treatments, start to manifest also in people with subclinical eating disorders (ED) symptomatology. Therefore, we propose a protocol for an exploratory experimental study to investigate whether participants with subclinical ED symptomatology and control participants differ in their performance on several eye movement tasks.

**Methods:**

The sample will be recruited through convenience sampling. The Eating Disorder Examination Questionnaire will be administered as a screening tool to split the sample into participants with subclinical ED symptomatology and control participants. A fixation task, prosaccade/antisaccade task, and memory-guided task will be administered to both groups. Additionally, we will measure anxiety and premorbid intelligence as confounding variables. Means comparison, exploratory Pearson's correlations and discriminant analysis will be performed.

**Discussion:**

This study will be the first to elucidate the presence of specific eye movement abnormalities in participants with subclinical ED symptomatology. The results may open opportunities for developing novel diagnostic tools/therapies being helpful to the EDs research community and allied fields.

## Background

Eating disorders (EDs) such as anorexia or bulimia nervosa are a serious problem in our society. They are relatively common, especially among young-adult women [1, 2]; are associated with severe quality-of-life impairments [3], medical and psychopathological comorbidities [4]; have the highest mortality rates of all psychiatric illnesses [5]; and require their own specialized treatments, which results in elevated care costs [6]. Further, EDs are an underestimated problem since a big part of the adult outpatients with EDs do not fully meet DSM criteria [7] and, despite advances in clinical research on EDs, a subset of patients does not improve after receiving treatment [8]. Accordingly, efficacious treatment for EDs is therefore of high importance.

In this context, a vast body of evidence indicates that an earlier diagnosis is considered essential to get more favourable therapeutic outcomes and cost-effective treatments [9–11]. Notwithstanding such advances in prevention research, a challenge reported in ED field is that somatic and psychosocial symptoms, as well as their comorbidities, can be difficult to detect in routine clinical care hindering their preventive treatment [12, 13]. On the other hand, individuals with EDs may fail to report their psychological symptoms [14], experience feelings of stigma and shame about seeking help [15] or have poor insight into perceiving the severity of the illness [14, 16].

To overcome this critical situation, a growing body of literature is focusing on the identification of distinctive biomarkers and endophenotypes associated with EDs [17, 18]. In broad terms, the term biomarkers refers to objectively measured indicators that indicate biological processes [19], while the term endophenotypes refers to quantitative traits in the causal pathway from the genotype to the phenotype [19]. Endophenotypes are distinguished from biomarkers by their causative role. On the contrary, biomarkers are just risk indicators of biological processes and do not play a causal role [19]. Despite their substantive differences, together, biomarkers and endophenotypes offer the potential to improve population-based screens for identifying people who are at risk for psychopathological disorders and can benefit from preventative treatments [20, 21].

While the findings should be interpreted with caution, some authors have speculated that EDs may be linked to a variety of biomarkers and endophenotypes, such as specific metabolic components [22], altered inner body perceptions [23, 24], or neuropsychological weaknesses (e.g., cognitive inflexibility or weak central coherence [25]). More specifically, studies using eye-tracking point out a possible attentional bias linked to disorder-related stimuli, such as food or bodies [26–28]. However, few eye-tracking studies have addressed the role of saccadic eye movements using non-disorder-related procedures (e.g., fixation or pro-/anti-saccade tasks). For example, a recent systematic review of the topic found 28 studies using disorder-related stimuli and only three were involved in the study of saccadic eye movements [28]. Paradoxically, this trend contrasts with the wide body of evidence in psychiatric research that suggests that specific disorders (e.g., depression or schizophrenia) are accompanied by specific patterns of saccadic abnormalities [29, 30]. As a result, in methodological terms, the use of non-disorder-related procedures aimed to identify saccadic eye movement abnormalities is a potential avenue for further research in the ED field. This is because it opens the possibility to provide objective tests and, ultimately, to improve prognostic and preventive treatments, which is an urgent call in the field as previously stated.

In this regard for example, Phillipou et al. [31] found that patients with AN showed poor performance on ocular fixations tasks, i.e., an impaired ability to maintain fixation on a single dot for a longer time compared with control participants. Specifically, they showed an increased rate of saccadic intrusions named square wave jerks (SWJs; these are horizontal, involuntary, saccadic intrusions that interrupt fixation). Recently, Phillipou et al. [32] identified the state independence and heritability of this possible biomarker. According to the results, they found that patients with AN, patients’ weight-restored from the illness, and sisters of people with AN, made significantly more SWJs than healthy controls. Furthermore, the combination of SWJ rate and anxiety showed high accuracy levels to discriminate between the different groups tested (≥ 70%). Another study using prosaccade/antisaccade and memory-guided saccade tasks showed the existence of specific eye movement anomalies in patients with AN [33]. The prosaccade task is stimulus-driven as it requires to perform a saccade (i.e., a ballistic movement of the eyes that shifts the centre of gaze) to an onset peripheral stimulus, a process which is difficult to inhibit. On the contrary, the antisaccade task is goal-driven since it requires volitional processing, a voluntary saccade to the opposite direction of the stimulus. The memory-guided saccade task requires a saccade towards a remembered onset peripheral stimulus after a brief delay. Compared to control participants, patients with AN tended to show shorter saccade latencies in the prosaccade/antisaccade task and more inhibitory errors in the memory-guided saccade task [33].

In sum, the potential clinical value of this research is noteworthy since it may allow screening people at risk of developing AN [18, 34]. The aforementioned research has placed a great focus on clinical and recovered AN samples. Nevertheless, there is a call to focus not only on AN samples, but also including diverse samples such as other ED subtypes [18]. According to this transdiagnostic perspective, it would be possible to find potential biomarkers or endophenotypes to identify "particular symptoms across heterogeneous illness profiles” [18]. In this context, it is not yet known whether these eye movement patterns start to manifest also in heterogeneous samples with subclinical ED symptomatology. Furthermore, studies using subclinical ED samples could help to disentangle a possible influence of starvation (which presumably is less extended under this population than in clinical ED) on these eye-movement patterns and shed light on whether these eye movement abnormalities may be a reflection of the condition itself. On the other hand, given the relative novelty of these results, there is a call in the field to replicate this underpinning research [18, 28], as well as to standardize some of these eye movement tasks (i.e., prosaccade/antisaccade tasks) [35].

### Study objectives and hypothesis

As Malcom and Phillipou [18] noted in their recent review, demonstrating a relationship with illness is one of the first steps in determining biomarkers and endophenotypes. As illustrative example, first Phillipou et al. [33] showed the existence of specific eye movement anomalies in patients with AN and only after these findings, they amplified the scope of their study and were able to identify the state-independence of these eye movement abnormalities [32]. Following this line of reasoning, we will investigate whether the aforementioned eye movement abnormalities detected are, in this case, associated in young-adult women with subclinical symptomatology. Therefore, this study will take as primary aim to investigate whether participants with subclinical ED symptomatology and control participants differ in their performance on a range of eye movement tasks. We hypothesize that participants with subclinical ED symptomatology compared to controls would be prone to show shorter saccade latencies and more inhibitory errors in a prosaccade/antisaccade task and in a memory-guided saccade task, respectively, and an increased rate of SWJs in a fixation task. Additionally, as secondary aim, and following the same approach of Phillipou et al. [31, 32], we will test the potential role of SWJ rate and anxiety for discriminating the different groups tested in our study (participants with subclinical ED symptomatology vs. control participants).

## Methods/design

### Participants and procedure

A young adult female sample will be chosen due to the higher prevalence of EDs in this population [1, 2, 7]. Thus, the sample will comprise young adult women in the 18–25 age range. The sample procedure will be through public advertisements and social media posts (convenience sampling). Inclusion criteria will be: (a) normal (or corrected) visual acuity; (b) gender: women; (c) current body mass index (BMI) in the normal range according to World Health Organization (WHO) (between 18.5–24.9). Exclusion criteria: (a) self-reported lifetime history of significant brain injury, neurological condition, ocular and/or visual pathology; (b) self-reported lifetime history of an ED or other mental illness; (c) self-reported current use of psychotropic drugs (e.g., antidepressants) or intake of recreational synthetic or natural drugs; (d) incomplete data collection or eye-tracker calibration failure; (e) inability to understand Spanish; (f) out of range age. The Spanish Eating Disorder Examination Questionnaire (S-EDE-Q) [36] will be administered since it is considered the gold standard for assessment of ED pathology [37]. The EDE-Q have shown good predictive as well as concurrent validity [37, 38]. Further, has been shown good psychometric properties in young adults in Spain [36]. This tool will serve as a pre-screening tool, allowing us to split the sample into two groups of participants with subclinical ED symptomatology and control participants according to their EDE-Q score, as defined by the study criteria (see below). A global EDE-Q score ≥ 2.8 has been shown to provide an optimal trade-off between sensitivity and specificity when it is used for screening in primary care [38]. The pre-screening will allow to form two groups of similar size: once the desired participant sample size has been reached for one of the groups, only participants falling into the other group defined for the study will be invited to take part.

At the beginning of the experiment a written informed consent will be obtained from all participants. Then, sociodemographic data collection and confounding variables like anxiety and premorbid intelligence quotient (IQ) will be measured. The instructions and questionnaires will be administered by a postgraduate student trained by senior members of the research team. Next, a battery of eye movement tasks presented in the same order will be administered (see the Measures section for more details). After completing the study, a debriefing session will be carried out. The full procedure will take approximately 90 min.

## Measures

### Psychometric measures

#### Eating disorder symptomatology

*Spanish Version of the Eating Disorder Examination Questionnaire (S-EDE-Q)* [36]. Composed by 28 7-point Likert-type response items ranging from 0 (not at all) to 6 points (markedly). Four subscales are measured, including dietary restraint, shape concerns, weight concerns, and eating concerns. The global score ranges from 0 to 6 points. As in Mond et al.’s [37], we will use a cut-off point ≥ 2.8 as clinically significant. Participants will be grouped according to this global index score.

To identify possible confounders, we will explore variables that may affect performance in eye movement tasks (premorbid intelligence and anxiety) [33]. Only those with significant relationships between groups will be deemed potential confounders and included in the analysis.

#### Premorbid intelligence

*The Word Accentuation Test* [39]. Composed by 30 low frequency Spanish words whose accents have been removed. Participants must demonstrate their knowledge of the correct accentuation of each word. The total score is the number of words correctly read (from 0 to 30). The test is administered individually and takes 2–3 min.

#### Anxiety levels

*State-Trait Anxiety Inventory (STAI)* [40]. The two forms of anxiety (state and trait) are separated in the inventory, and both have their own 20 separate questions. The questionnaire is therefore composed of 40 items ranging from 0 (almost never) to 4 (almost always). The score ranges from 0 to 80 points. For both forms, higher scores indicate higher levels of anxiety.

### Eye movement measures

#### Data acquisition

Participants will sit in a bright room with constant lighting and temperature in front of a 24″ LED 144 Hz monitor (Asus VG248QE; 60-Hz refresh rate) with resolution 1920 × 1080 pixels. An adjustable chin rest will be used to minimize head movements and to ensure a constant distance between the participants’ eyes and the screen (90 cm). Eye movements will be recorded with the EyeLink Portable Duo (SR Research, Ontario, Canada). Both eyes will be recorded at a sampling rate of 500 Hz. The device uses a dark pupil-to-cornea reflection method. A saccade velocity threshold of 30°/sec, an acceleration threshold of 8000°/s^2^ and a motion threshold of 0.15° will be set. Before each task, an automatic randomized 9-point calibration will be conducted on a black background screen and drift correction will be performed throughout the task when necessary.

#### Eye movement tasks

**Fig. 1:**
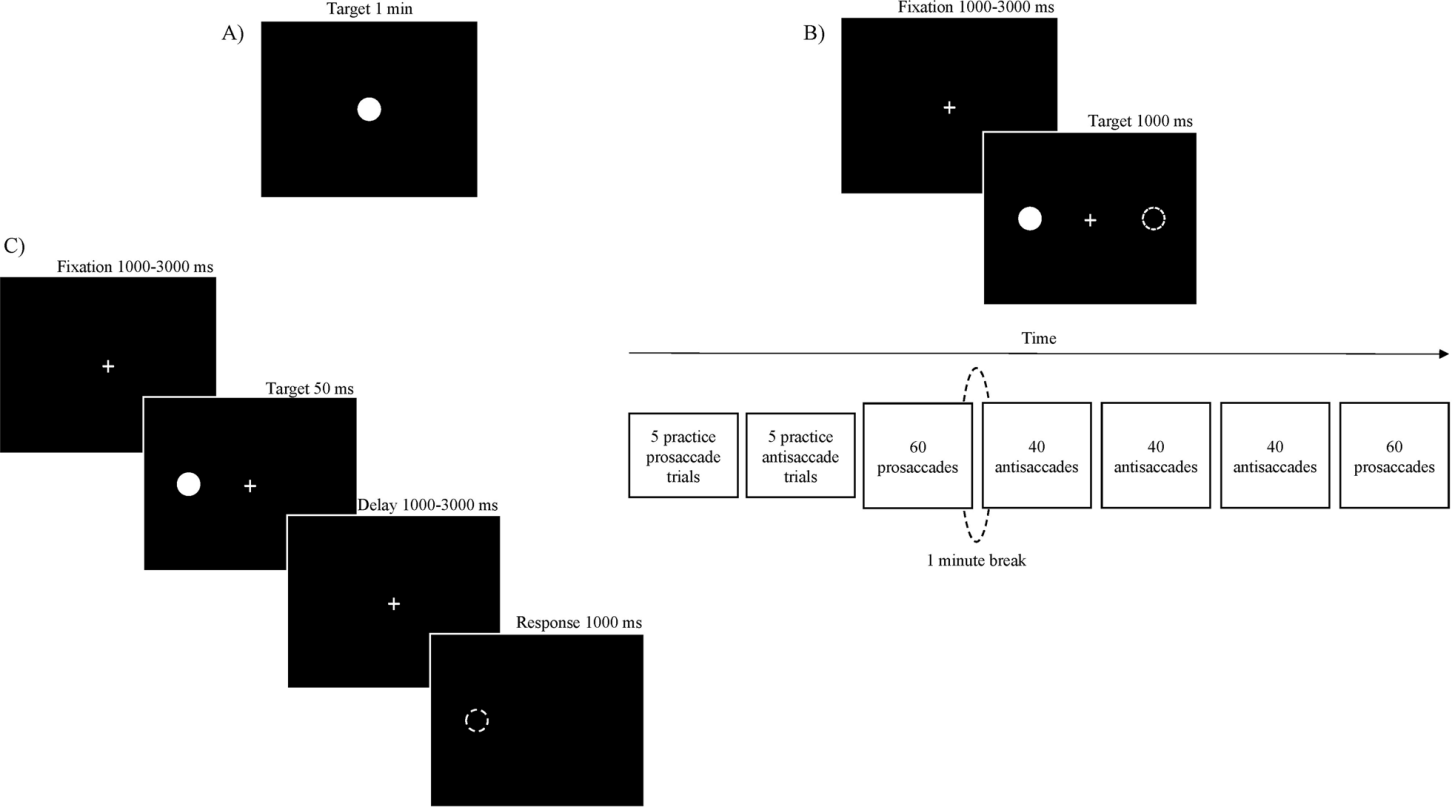
Eye-tracking tasks. **a** Fixation task, **b** Prosaccade/Antisaccade task, **c** Memory-guided task

*Fixation task* [31]. The participant is asked to look at a centred fixation dot (diameter of 0.5 degrees) for 1 min (Fig. 1a). The task comprises three trials with a short resting period between them.

*Prosaccade/antisaccade task* [35]. Each trial begins with a centred fixation cross (random period 1000–3000 ms) followed by a dot (diameter of 0.5 degrees) that appears in a random location either 8º left or right of the fixation cross (1000 ms). In the prosaccade condition the participant is required to direct her gaze towards the dot as quickly as possible, while in the antisaccade condition the participant is required to direct her gaze to the opposite position of the dot (Fig. 1b). First, participants are required to complete a practice phase consisting of five prosaccade trials and five antisaccade trials. Once practice trials are completed, a total of 240 trials are presented organized in five blocks as follows: 60 prosaccades; 40 antisaccades; 40 antisaccades; 40 antisaccades; 60 prosaccades (Fig. 1b).

*Memory-guided saccade task* [33]. The participant is asked to look at a centred fixation cross for a random period from 1000 to 3000 ms. Next, a 50 ms-dot (diameter of 0.5 degrees) is presented in a random location in the periphery (5–10° left or right) while the central fixation remains for a random period in the interval 1000–3000 ms. The participant is instructed to keep her eyes on this central fixation during the entire delay and to trigger a saccade towards the memorised location of the peripheral dot as soon as the central fixation disappears. First, participants are required to complete a practice phase consisting of eight trials. Once practice trials are completed, a total of 52 trials are presented, with an equal number of target presentations for each peripheral location (Fig. 1c).

For all the tasks: cross fixation colour, font type and size (RGB: 255,255,255; Font: Times New Roman, 28). Background screen colour (RGB: 51,51,51).

### Other measures

Age, socioeconomic status, and highest level of education completed will be collected as sociodemographic variables. BMI will be measured and computed according to population norms and adjusted for age, sex, and height.

### Data analysis

Eye-tracking data will be analysed with SR Research’s program DataViewer and Microsoft Excel. See Table 1 for a detailed overview of the outcome measures associated with the eye-tracking data. The proportion of participants and excluded trials, and the percentage of lost data will be reported.

**Table 1:** Outcome measures associated with the eye-tracking data

Task	Outcomes		
Fixation	Rate of SWJs. Threshold for SWJ detection included saccade pairs (an initial saccade that moves the fovea away from the cross fixation, followed by a second saccade in the opposite direction to refoveate the fixation) within 200 ms, with amplitudes between 0.1° and 5°		
Prosaccade	Gain, latency, and peak velocity of correct saccades	Errors	*Anticipation rate* making a saccade during the fixation period, prior to the presentation of the target dot or within 80 ms of its presentation
			*Prosaccade error* saccade in the opposite direction of the peripheral stimulus
Antisaccade	Gain, latency, and peak velocity of correct saccades	Errors	*Corrected error rate* looking at the target, then looking at the correct location at the mirror image of the target
			*Latency of the correction saccade* how long it took to make a saccade in the correct direction following the incorrect saccade
			*Uncorrected error rate* looking at, instead of away from, the target
			*Anticipation rate* making a saccade during the fixation period, prior to the presentation of the target dot or within 80 ms of its presentation
Memory-guided saccade	Gain, latency, and peak velocity of correct saccades	Errors	*Inhibitory errors* Looking at the dot when it was presented or making a saccade before the response period
			*Directional errors* Looking in the wrong direction to where the stimulus had been presented during the response time

Practice trials are aimed to acquaint the participant with the experimental paradigms and will not be statistically analyzed. In the saccade tasks, saccades smaller than 2° will not be analyzed. The error rate for each participant will be calculated as the proportion of erroneous trials to all valid trials. The gain of the first saccade will be calculated as a ratio of the first saccade amplitude divided by the desired saccade amplitude (e.g., 8° for Prosaccade task). Latency of the first saccade is defined as the latency from appearance of the target to the start of the saccade

### Sample size calculation

An 80% of power analysis and a significant criterion of α = 0.05 were set since these are considered a convention for general use specifically in the psychology field [41, 42]. Regarding the effect size, based on previous literature on the topic [34], we choose a large effect size (d = 0.8) [41]. Thus, a priori t-test G*Power v3.1.9.2 (Dusseldorf University, Germany, http://www.gpower.hhu.de/en.html) analysis yields a minimum sample of 26 participants in each group (N = 52, two groups: participants with subclinical ED symptomatology vs. control participants). Nevertheless, if significant results were not obtained after completion of the study, a retrospective power analysis will be carried out with the participants responses to verify whether the non-significant result is due to lack of relationship between the groups or due to lack of statistical power. In this case, more participants will be recruited.

### Statistical analysis

Statistical analyses will be performed with SPSS v.28. Normality and outliers will be checked through graphic tests such as QQ plots, histograms, and box plots. Cronbach’s α for each questionnaire will be reported. Group analyses will be performed with Student's t-test paired comparison (for normal distribution) or Wilcoxon tests (for non-normal distribution). We will apply a correction for multiple t-test comparisons. Cohen’s d will be reported for variables showing significance. In case of significant differences between study groups, we will then include confounding variables (premorbid intelligence and anxiety) as covariates in the analysis described above. Across groups, exploratory Pearson's correlation analyses will also be conducted between S-EDE-Q, STAI scores, and BMI and the outcome measures associated with the eye-tracking data (see Table 1, for a detailed view). Additionally, if significant differences are found between STAI scores and SWJ rate, consistent with previous literature [31, 32], a discriminant analysis will be performed on the entire sample to elucidate to what extent both measures correctly classify group membership (participants with subclinical ED symptomatology vs. control participants). Alpha will be set at 0.05 for all analyses.

## Discussion

Basic research using non-disorder-related eye-tracking procedures indicates that patients with AN show specific eye movement abnormalities. Taken together, these results may indicate the existence of specific eye-tracking measures that could be used as screening tools of people at risk of AN, although replications with larger sample sizes are needed. Therefore, the upshot of this promising line of research may help to deepen our understanding of the nature of these eye-movement abnormalities as potential biomarkers and endophenotypes to develop future preventive treatments. In our view, exploring the presence of these patterns of eye movements in a subclinical ED phase is also crucial since once individuals develop an ED, it might be more difficult, or even too late, to improve treatment outcomes. Thus, our exploratory research aims to extend previous literature to shed new light on whether the presence of distinctive eye movement patterns is also present in participants with subclinical ED symptomatology. Additionally, we will test the accuracy of SWJ rate and anxiety to discriminate the different groups tested (participants with subclinical ED symptomatology vs. control participants). The findings may be the starting point to lay the basis for future studies aimed to clarify the state-indepence or the heritability of these eye movements abnormalities in subclinical populations. By doing so, the existence of putative biomarkers and endophenotypes under this population could be argued with a higher degree of evidence.

## Data Availability

The datasets generated and/or analysed during the current study will be available in the Open Science Framework.

## Abbreviations

EDs: Eating disorders
AN: Anorexia nervosa
SWJs: Square wave jerks
BMI: Body mass index
IQ: Intelligence quotient
WHO: World Health Organization

## Declarations

### Study status

Data collection will be in February 2022.
